# Supplementary material for: Multiple sources of aerobic methane production in aquatic ecosystems include bacterial photosynthesis
Source: Nat Commun. 2022 Oct 29;13:6454. doi: 10.1038/s41467-022-34105-y (PMC9617973; doi:10.1038/s41467-022-34105-y)
Supplement: Supplementary file 1 — Supplementary Information [file 41467_2022_34105_MOESM1_ESM.pdf]

Supplementary Information for

Multiple sources of aerobic methane production in aquatic ecosystems include bacterial photosynthesis

Elisabet Perez-Coronel and J. Michael Beman

This PDF file includes:

Supplementary Notes 1-3

Figures S1 and S2

Tables S1 to S7

Supplementary References

Supplementary Note 1

Methane production and consumption over time

All experiments were conducted for at least 24 hours to permit direct comparisons across experiments. However, we included multiple sampling time points before 24 hours in some experiments, while others were conducted for up to 96 hours (Table S2). Although longer incubations may introduce bottle effects, this has been used in earlier work[1,2], and provides multiple comparisons through time, between treatments, and across experiments.

In experiments with multiple sampling time points before 24 hours, we occasionally observed initial decreases that were followed by increases by 24 hours. This could be explained by the development of P limitation that triggers methane (CH_4) production; for example, Karl et al.[3] observed CH_4 production by MPn breakdown only under P-stressed conditions. Other incubations showed the opposite pattern: an initial increase followed by a decrease in concentration (L2, UC2, LG1). Longer experiments likewise showed eventual decreases in CH_4 concentrations after 24 hours (e.g., L4, L7, LC1, UC4). This behavior has been observed in earlier studies[4] and was the intention of these experiments; specifically, longer incubations may capture the time lag between initial production followed by eventual consumption once CH_4 concentrations exceed a required threshold for methanotrophy. Put another way, both CH_4 production and consumption are taking place, but production rates exceed oxidation rates until oxidation 'catches up' and eventually exceeds production. In general, *pmoA* transcripts were more abundant when CH_4 concentrations in the incubations were higher (L7, LG5 and LG6; CH_4 >100 nM)—consistent with threshold-dependent CH_4 oxidation[4]. A similar pattern was evident in metagenomes, as *pmoA* genes were absent in the LG2 incubations where the CH_4 concentrations were <50 nM, and in low abundance in the L6 incubations (CH_4 = ~100 nM).

Supplementary Note 2

Additional significantly different functional families in experimental treatments

In addition to DPOR and COR, two functional families were also significantly higher ($P<0.05$) in both the BES and high light intensity treatments in the UC4 experiment. One of these, magnesium chelatase, is also involved in (bacterio)chlorophyll synthesis, where it catalyzes the insertion of a magnesium atom. Geranylgeranyl diphosphate reductase was also higher in the BES and high-light treatments; this can play several roles, including a role in

(bacterio)chlorophyll biosynthesis[5]. Magnesium chelatase expression increased 304% in the BES treatment and 107% under high light intensity, whereas geranylgeranyl diphosphate reductase expression increased 433% and 37%, respectively. Beta-glucosidase expression was also significantly higher in the BES treatment but not under high light, while several functions (including several that are photosynthesis-related) were higher under high light intensity but not in the BES treatment: magnesium-protoporphyrin IX monomethyl ester (oxidative) cyclase, NADH dehydrogenase, non-specific serine/threonine protein kinase, proton-exporting ATPase, and RNA helicase.

Supplementary Note 3

Effects of BES and other sulfonates

Earlier work indicates four or more possibilities for how BES may affect aquatic microorganisms and subsequent CH_4 production. First, no inhibitor is totally specific[6], and although BES is effective at inhibiting methanogenesis, it is known to disrupt multiple groups of organisms and pathways beyond methanogenesis[6-14]. Because BES is rarely applied to oxygenated samples and ecosystems, it may have additional unrecognized effects on aerobic organisms or pathways, possibly including aerobic photosynthetic organisms. Second, other sulfonates are known to have a variety of environmental effects that may also apply to BES, but also remain undocumented. For example, additions of perfluorooctane sulfonate drove the formation of reactive oxygen species by the green algae *Chlorella vulgaris*[15]. Third, sulfonates are actively cycled in surface waters of the ocean, where they are produced by phytoplankton and subsequently metabolized by other abundant microorganisms[16]. Interestingly, several of the enzymes involved in sulfonate metabolism are related to those used in methanogenesis, and are thought to act promiscuously on many compounds[16-20]. The underlying reason for production of sulfonates by marine phytoplankton remains unknown, but production is linked to the diel light cycle, and may be related to maintenance of redox balance[16]. In our BES treatments, addition of a sulfonate in comparatively high concentrations may have potentially interfered with sulfonate production or metabolism in (freshwater) photosynthetic microorganisms. Finally, the simplest possibility is that addition of an artificial compound in comparatively high concentrations acts as a stressor that drives a common response. This may be analogous to CH_4 production in terrestrial plants, where very different external stressors (e.g., changes in temperature, light, physical damage, etc.)

all similarly result in CH_4 production[21-24]. This is thought to be regulated by a common mechanism of oxidative stress[24]. Given its evident importance in plant CH_4 emissions, oxidative stress may also be relevant for aquatic photosynthetic organisms, and could be examined following the approaches used in previous plant-based research[25]. In our experiments, the fact that BES and high light intensity produced surprisingly similar patterns in CH_4 production, $\delta^{13}CH_4$ values, and gene expression (Figs. 3-5) suggests a common response mechanism that leads to CH_4 production. BES may provide another way to study and understand this response.

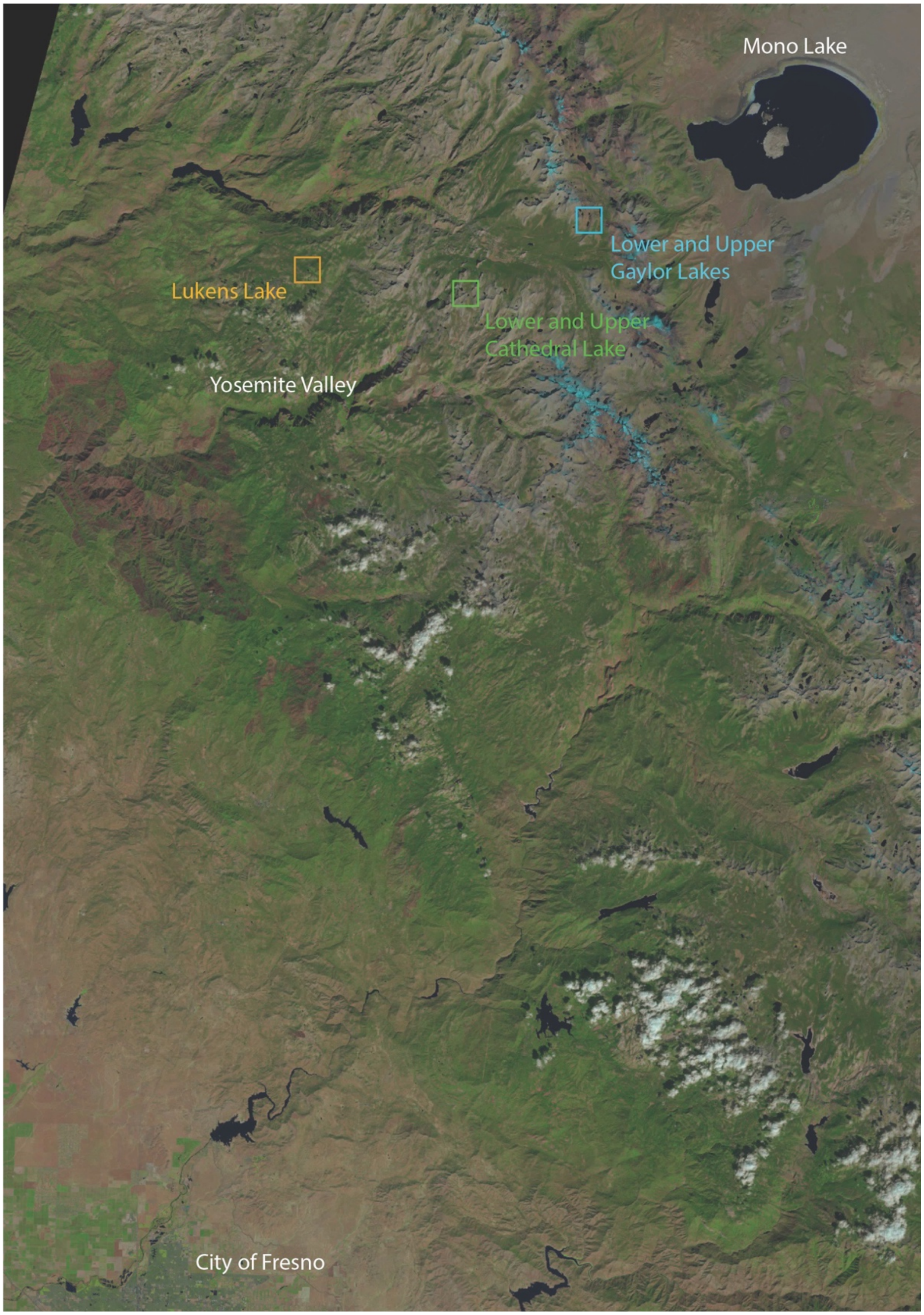

Figure S1. Sampling locations in Yosemite National Park displayed on a Landsat 8 image of the region collected on October 9th, 2018 (path 42, row 34; Landsat imagery courtesy of NASA Goddard Space Flight Center and U.S. Geological Survey). Lukens Lake is located farthest west and the Gaylor Lakes farthest east, with the Cathedral Lakes midway between. Locations of Mono Lake, Yosemite Valley, and the City of Fresno are also shown.

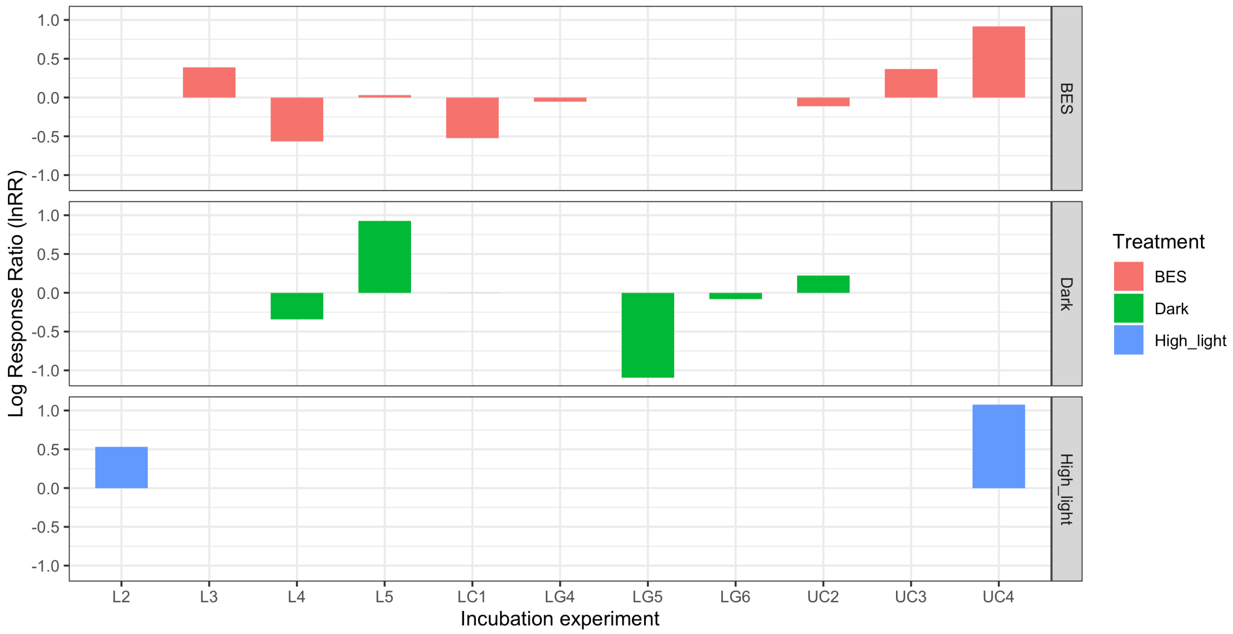

Figure S2. Log response ratios for methane production rates in experimental treatments. Data are for comparisons with controls at the end of the experiments, and are only shown where experimental treatments elicited a significant effect. Experiments L3, L5, LG5 and UC3 were longer experiments and treatment effects are shown for 36, 74, 86 and 57 hours respectively (all others are shown at 24 hours).

Table S1. Summary of lake characteristics, including dissolved oxygen values during experiments and mean carbon and nutrient concentrations.

	**Lukens Lake**	**Lower Cathedral Lake**	**Upper Cathedral Lake**	**Lower Gaylor Lake**	**Upper Gaylor Lake**
Elevation (m)	2489	2815	2905	3115	3185
Mean temperature during sampling (°C)	17.2	16.7	17.4	13.8	12.8
Mean initial experimental dissolved oxygen (mg L^{-1})	6.7	7.53	9.7	9.7	5.3
Mean final experimental dissolved oxygen (mg L^{-1})	5.8	8.15	8.5	7.2	5.2
Mean dissolved organic carbon (mg L^{-1})	5	1.9	1.8	1.7	1
Mean dissolved nitrite (µM)	0.2	0.2	0.2	0.2	0.2
Mean dissolved nitrate (µM)	0.3	0.5	0.3	0.6	0.4
Mean dissolved ammonium (µM)	0.7	0.8	0.7	1.3	0.8
Mean dissolved phosphate (µM)	0.2	0.1	0.1	0.3	0.2

Table S2. Details of experimental incubations, including: experiment number; year, month, and days of experiment; whether methane production or oxidation was significant in any of the controls or treatments; generation of 16S rDNA, 16S rRNA, metatranscriptomes, and metagenomes; length of experiment; and treatments. BES corresponds to the addition of 2-bromoethanesulphonate as a methanogenesis inhibitor, Dark represents experiments incubated in total darkness and HL (high light) are experiments subjected to high light intensity during the incubation.

**Experiment**	**Year**	**M**	**Days**	**CH_4**	**16S rDNA**	**16S rRNA**	**Metatranscriptome**	**Metagenome**	**Length (hr)**	**Treatments**
L1	2016	8	12-13	+	X				24	
L2	2016	9	11-12	+	X				24	HL
L3	2017	7	28-30	+	X				48	BES, Dark
L4	2017	8	8-10	-	X				48	BES, Dark
L5	2017	9	26-29	+		X			74	BES, Dark
L6	2017	11	13-15		X	X	X	X	55	BES
L7	2018	9	23-28	-		X	X		96	BES, Dark, HL
LG1	2016	8	8-9	+	X				24	
LG2	2016	9	9-10		X			X	24	
LG3	2016	10	7-8	+	X				24	
LG4	2017	8	24-26	+	X				48	BES, Dark
LG5	2017	10	1-4	-		X	X		86	BES, Dark
LG6	2018	9	10-14	-		X	X		96	BES, Dark, HL
UC1	2016	8	10-11	+	X				24	
UC2	2017	8	10-12	+					48	BES, Dark
UC3	2017	10	8-11	+		X			57	BES, Dark
UC4	2018	10	8-12	+		X	X		96	BES, Dark, HL
LC1	2017	8	26-28	-/+					48	BES, Dark
UG1	2017	8	12-14						48	BES

Table S3. Percentage of *phnJ*, DPOR, and COR transcripts and genes, as well as the mean and range of percent identity (ID%), for significant microbial taxa in metatranscriptomes and metagenomes. Blank cells indicate that a particular group is irrelevant for that particular gene.

	Comamonadaceae	**Cyanobacteria**	***Polynucleobacter***	**Other *Burkholderiales***
% of *phnJ* transcripts	20.9			
phnJ transcripts mean (and range) ID%	92.2 (69.6-100)			
% of *phnJ* genes	27.4			
phnJ genes mean (and range) ID%	84.8 (60.4-100)			
% of DPOR transcripts	30.6	6.3	5.7	2.8
DPOR transcripts mean (and range) ID%	88.8 (60.4-100)	91.5 (63.5-100)	94.5 (69.7-100)	92.1 (60.2-100)
% of DPOR genes	25.7	0.3	35.3	1.4
DPOR genes mean (and range) ID%	86.2 (60-100)	86.9 (64.4-100)	89.8 (60-100)	88.2 (60-100)
% of COR transcripts	48.7		5.8	6.8
COR transcripts mean (and range) ID%	91.5 (60-100)		94.3 (61.4-100)	93.6 (61.7-100)
% of COR genes	29.8		34.1	2.4
COR genes mean (and range) ID%	88.5 (60-100)		90.7 (60-100)	87.3 (60-100)

Table S4. Aspartate aminotransferase (*aat*) transcripts or gene sequences (as a percentage of *recA* transcripts or genes) from relevant freshwater bacterial groups in metatranscriptomes and metagenomes.

**Metatranscriptome/ metagenome**	***Acidovorax***	***Pseudomonas***	***Caulobacter***	***Mesorhizobium***	***Polaromonas***	***Limnohabitans***
LG5	0.24	0.00	0.03	0.10	0.90	2.66
LG6	0.07	0.00	0.71	0.00	0.39	1.29
L7	0.14	0.00	0.07	0.00	0.07	1.69
UC4 Control	0.31	0.12	1.28	0.00	2.44	5.80
UC4 BES	0.25	0.10	1.64	0.00	1.44	3.67
UC4 High light	0.88	0.00	1.39	0.00	3.03	4.05
LG2 t0	0.43	0.04	2.37	0.00	2.40	4.77
LG2 tf	0.34	0.02	3.48	0.00	2.22	6.85
L6 t0	0.14	0.08	0.06	0.00	0.26	1.92
L6 tf	0.14	0.02	0.09	0.02	0.25	1.46
L6 BES	0.15	0.00	0.02	0.00	0.09	1.75

Table S5. Percent identity of 16S rRNA sequences from abundant *Comamonadaceae* ASVs to those in sequenced genomes in the RefSeq Genome Database.

ASV	%ID	Genome
b66e24bf473f06a1fc27fa300549636c	99.7	Limnohabitans Rim47 and others, Acidovorax radicis and others
14f5d35b60a4aa29fb4630dc0531b12e	99.1	Acidovorax temperans, delafieldii and other strains
f361f2ed2d8297ddacd4e36526eb8265	100	Limnohabitans Rim11
f2fce6a8c3a54fda2ae66977fd9b45af	99	Limnohabitans Rim11 and MMS10
222b12c7663803b96339c5af33e2b3ae	100	Limnohabitans 103DPR2
9b6d5192b8dc30e78ce626298c8a0a26	99.4	Limnohabitans Rim47 and others, Acidovorax radicis and others
95dd5f1247bb97a138d1faf91e409f85	99.3	Limnohabitans 103DPR2 and JirII
62767018de03c34a4305384df06ce393	98.8	Acidovorax delafieldii, others
86832a3826a81807da70809f71eed601	99.4	Limnohabitans Rim47 and others, Acidovorax radicis and others
5efb46c72256df114e8f2b354fb44192	99.7	Limnohabitans Rim11 and MMS10
d81e17e704250a1f80e1005be449b274	99.4	Limnohabitans 103DPR2 and JirII
12e95e1ebf7046141228b71394bd745b	99.7	Limnohabitans planktonicus, Rim28, and others
02523daf5356885c7d28e05d5910acb3	100	Rhodoferax sediminis, ferrireducens, saidenbachensis, others
8f8863b1fba0419252ce1756ec2f9b46	100	Rhodoferax bucti strain GSA243-2
1b24e60fca2c1ce5d0e34cab586c4537	100	Polaromonas sp. YR568
d20e921d6be99c2d20fe3d688f878774	99.7	Rhodoferax bucti strain GSA243-2
74dace2780cac91c5a0d5598db981acc	100	Limnohabitans Rim47 and others, Acidovorax radicis and others
6f6aa478c9f6ad0d6f8b0a415706fa81	100	Limnohabitans curvus, others
317124a0271aaaea477061ada50e0cfb	99.4	Limnohabitans Rim8, others
c4143240980a12d00bf11daf4fefbd10	99.7	Limnohabitans Rim8, others
12fd09c14da54c5c7c412f7e493c4e16	100	Limnohabitans planktonicus, others
c4d004171ee776f1e529ea5dcb010508	100	Rhodoferax koreense strain DCY-110, Curvibacter sp. AEP1-3
405bafe3cfaf0d7ba859b721abc30c01	98.8	Comamonas terrigena (multiple strains)
1f796798ba249fd048e233237b86bf05	98.8	Pelomonas (multiple)

Table S6. Examples of multiple *phn* genes co-located on assembled contigs (>10,000 base pairs in length) within metagenomes from the LG2 and L6 experiments.

**Exp.**	**Contig**	**bp**	***phnV***	***phnO***	***phnN***	***phnM***	***phnL***	***phnK***	***phnJ***	***phnI***	***phnH***	***phnG***	***phnF***	***phnE***	***phnD***	***phnC***
LG2	k141_33043	23126		1	1	1	1	1	1	1				1	1	1
LG2	k141_67057	20078			1	1	1	1	1	1	1	1				1
LG2	k141_174869	50613			1	1	1	1	1	1	1	1	1	1		
LG2	k141_61413	94539			1		1	1	1	1	1	1		1		1
LG2	k141_68876	34960			1	1	1	1	1	1	1	1	1	1		1
L6	k141_232042	14329	1						1	1	1	1				
L6	k141_58845	164079	1				1	1	1	1	1	1		1		
L6	k141_137475	51136			1		1	1	1	1	1	1			1	1

Table S7. List of betaproteobacterial metagenome-assembled genomes (MAGs) and links to scaffolds containing COR, DPOR, and phosphonate metabolism genes in the IMG database.

MAG	Affiliation	COR gene scaffold(s)	DPOR gene scaffold(s)	Phosphonate metabolism genes scaffold(s)
1	*Rubrivivax*	Ga0136641_1000428	Ga0136641_1000214	Ga0136641_1000005<br>Ga0136641_1000835
5	*Limnohabitans*	Ga0136641_1000705	Ga0136641_1000769<br>Ga0136641_1001408	Ga0136641_1001116<br>Ga0136641_1001732
6	*Limnohabitans*	Ga0136641_1000147	Ga0136641_1000390<br>Ga0136641_1003698	Ga0136641_1000400<br>Ga0136641_1004149
18	*Limnohabitans*	Ga0136642_1003267		Ga0136642_1004820
20	*Polynucleobacter*	Ga0136642_1002146	Ga0136642_1003437	

Supplementary References

1. M. Yao, C. Henny, J. A. Maresca, Freshwater Bacteria Release Methane as a By-Product of Phosphorus Acquisition. *Appl Env. Microbiol* **82**, 6994–7003 (2016).

2. Q. Wang, J. E. Dore, T. R. McDermott, Methylphosphonate metabolism by Pseudomonas sp. populations contributes to the methane oversaturation paradox in an oxic freshwater lake. *Environ. Microbiol.* **19**, 2366–2378 (2017).

3. D. M. Karl, *et al.*, Aerobic production of methane in the sea. *Nat. Geosci.* **1**, 473–478 (2008).

4. H.-P. Grossart, K. Frindte, C. Dziallas, W. Eckert, K. W. Tang, Microbial methane production in oxygenated water column of an oligotrophic lake. *Proc. Natl. Acad. Sci.* **108**, 19657–19661 (2011).

5. Proctor, M. S., Sutherland, G. A., Canniffe, D. P., & Hitchcock, A. The terminal enzymes of (bacterio) chlorophyll biosynthesis. *Royal Society Open Science* **9**, 211903 (2022).

6. Oremland, R. S. & Capone, D. G. Use of "Specific" Inhibitors in Biogeochemistry and Microbial Ecology. in *Advances in Microbial Ecology* (ed. Marshall, K. C.) 285–383 (Springer US, 1988). doi:10.1007/978-1-4684-5409-3_8.

7. Liu, H., Wang, J., Wang, A., & Chen, J. Chemical inhibitors of methanogenesis and putative applications. *Applied Microbiology and Biotechnology* **89**, 1333-1340 (2011).

8. Dicker, H. J., & Smith, D. W. Effects of organic amendments on sulfate reduction activity, H2 consumption, and H2 production in salt marsh sediments. *Microbial Ecology* **11**, 299-315 (1985).

9. Ye, D., Quensen III, J. F., Tiedje, J. M., & Boyd, S. A. 2-Bromoethanesulfonate, sulfate, molybdate, and ethanesulfonate inhibit anaerobic dechlorination of polychlorobiphenyls by pasteurized microorganisms. *Applied and Environmental Microbiology* **65**, 327-329 (1999).

10. Chidthaisong, A., & Conrad, R. Specificity of chloroform, 2-bromoethanesulfonate and fluoroacetate to inhibit methanogenesis and other anaerobic processes in anoxic rice field soil. *Soil Biology and Biochemistry* **32**, 977-988 (2000).

11. Chiu, P. C., & Lee, M. 2-Bromoethanesulfonate affects bacteria in a trichloroethene-dechlorinating culture. *Applied and Environmental Microbiology* **67**, 2371-2374 (2001).

12. Boyd, J. M., Ellsworth, A., & Ensign, S. A. Characterization of 2-bromoethanesulfonate as a selective inhibitor of the coenzyme M-dependent pathway and enzymes of bacterial aliphatic epoxide metabolism. *Journal of Bacteriology* **188**, 8062-8069 (2006).

13. Boyd, J. M., Clark, D. D., Kofoed, M. A., & Ensign, S. A. Mechanism of inhibition of aliphatic epoxide carboxylation by the coenzyme M analog 2-bromoethanesulfonate. *Journal of Biological Chemistry* **285**, 25232-25242 (2010).

14. Xu, K., Liu, H., & Chen, J. Effect of classic methanogenic inhibitors on the quantity and diversity of archaeal community and the reductive homoacetogenic activity during the process of anaerobic sludge digestion. *Bioresource Technology* **101**, 2600-2607 (2010).

15. Xu, D., Chen, X., & Shao, B. Oxidative damage and cytotoxicity of perfluorooctane sulfonate on chlorella vulgaris. *Bulletin of Environmental Contamination and Toxicology* **98**, 127-132 (2017).

16. Durham, B. P., *et al.* Sulfonate-based networks between eukaryotic phytoplankton and heterotrophic bacteria in the surface ocean. *Nature Microbiology* **4**, 1706-1715 (2019).

17. Denger, K. *et al.* Bifurcated degradative pathway of 3-sulfolactate in *Roseovarius nubinhibens* ISM via sulfoacetaldehyde acetyltransferase and (*S*)-cysteate sulfolyase. *J. Bacteriol.* **191**, 5648–5656 (2009).

18. Graham, D. E., Taylor, S. M., Wolf, R. Z. & Namboori, S. C. Convergent evolution of coenzyme M biosynthesis in the Methanosarcinales: cysteate synthase evolved from an ancestral threonine synthase. *Biochem. J.* **424**, 467–478 (2009).

19. Helgadóttir, S., Rosas-Sandoval, G., Söll, D. & Graham, D. E. Biosynthesis of phosphoserine in the Methanococcales. *J. Bacteriol.* **189**, 575–582 (2007).

20. Graupner, M., Xu, H. & White, R. H. Identification of an archaeal 2-hydroxy acid dehydrogenase catalyzing reactions involved in coenzyme biosynthesis in methanoarchaea. *J. Bacteriol.* **182**, 3688–3692 (2000).

21. Keppler, F., Hamilton, J. T. G., Braß, M. & Röckmann, T. Methane emissions from terrestrial plants under aerobic conditions. *Nature* **439**, 187–191 (2006).

22. Messenger, D. J., Mcleod, A. R. & Fry, S. C. The role of ultraviolet radiation, photosensitizers, reactive oxygen species and ester groups in mechanisms of methane formation from pectin. *Plant, Cell & Environment* **32**, 1–9 (2009).

23. Vigano, I. *et al.* Effect of UV radiation and temperature on the emission of methane from plant biomass and structural components. *Biogeosciences* **5**, 937–947 (2008).

24. Bruhn, D., Møller, I. M., Mikkelsen, T. N. & Ambus, P. Terrestrial plant methane production and emission. *Physiologia Plantarum* **144**, 201–209 (2012).

25. Hilt, S., Grossart, H. P., McGinnis, D. F., & Keppler, F. Potential role of submerged macrophytes for oxic methane production in aquatic ecosystems. *Limnology and Oceanography* https://doi.org/10.1002/lno.12095 (2022).
