# Supplementary material for: Multiple sources of aerobic methane production in aquatic ecosystems include bacterial photosynthesis
Source: Nat Commun. 2022 Oct 29;13:6454. doi: 10.1038/s41467-022-34105-y (PMC9617973; doi:10.1038/s41467-022-34105-y)
Supplement: Supplementary file 3 — Reporting Summary [file 41467_2022_34105_MOESM3_ESM.pdf]

nature portfolio

Corresponding author(s): J Michael Beman

Last updated by author(s): Oct 10, 2022

Reporting Summary

Nature Portfolio wishes to improve the reproducibility of the work that we publish. This form provides structure for consistency and transparency in reporting. For further information on Nature Portfolio policies, see our Editorial Policies and the Editorial Policy Checklist.

Statistics

For all statistical analyses, confirm that the following items are present in the figure legend, table legend, main text, or Methods section.

n/a	Confirmed	
☐	☒	The exact sample size (n) for each experimental group/condition, given as a discrete number and unit of measurement
☐	☒	A statement on whether measurements were taken from distinct samples or whether the same sample was measured repeatedly
☐	☒	The statistical test(s) used AND whether they are one- or two-sided<br>*Only common tests should be described solely by name; describe more complex techniques in the Methods section.*
☒	☐	A description of all covariates tested
☒	☐	A description of any assumptions or corrections, such as tests of normality and adjustment for multiple comparisons
☐	☒	A full description of the statistical parameters including central tendency (e.g. means) or other basic estimates (e.g. regression coefficient) AND variation (e.g. standard deviation) or associated estimates of uncertainty (e.g. confidence intervals)
☐	☒	For null hypothesis testing, the test statistic (e.g. F, t, r) with confidence intervals, effect sizes, degrees of freedom and P value noted<br>*Give P values as exact values whenever suitable.*
☒	☐	For Bayesian analysis, information on the choice of priors and Markov chain Monte Carlo settings
☒	☐	For hierarchical and complex designs, identification of the appropriate level for tests and full reporting of outcomes
☐	☒	Estimates of effect sizes (e.g. Cohen's d, Pearson's r), indicating how they were calculated

Our web collection on statistics for biologists contains articles on many of the points above.

Software and code

Policy information about availability of computer code

Data collection	None used
Data analysis	RStudio v1.2.5001, Divisive Amplicon Denoising Algorithm (DADA2) v1.12, QIIME2 v2019.7.0, mothur v1.42.2, BBDuk v38.23, PANDASeq v2.11, DIAMOND v0.9.30, megahit v1.1.3, Integrated Microbial Genomes & Microbiomes platform (IMG/M) abundance profile viewer, bowtie2 v2.3.4.3, samtools v1.11, MetaBAT v0.32.4 and 0.32.5, RefineM v0.0.20, Last v876, CheckM v1.0.11, 'segmented' package v1.3-4 in R; 'aov' and 'lm' functions in R; 'HSD.test' function from the 'agricolae' package v.1.3.3 in R.

For manuscripts utilizing custom algorithms or software that are central to the research but not yet described in published literature, software must be made available to editors and reviewers. We strongly encourage code deposition in a community repository (e.g. GitHub). See the Nature Portfolio guidelines for submitting code & software for further information.

Data

Policy information about availability of data

All manuscripts must include a data availability statement. This statement should provide the following information, where applicable:

- Accession codes, unique identifiers, or web links for publicly available datasets
- A description of any restrictions on data availability
- For clinical datasets or third party data, please ensure that the statement adheres to our policy

DNA and RNA sequence data from this study are available in the Sequence Read Archive under BioProject PRJNA656136 (https://www.ncbi.nlm.nih.gov/bioproject/PRJNA656136). CH_4 concentration data (https://datadryad.org/stash/dataset/doi:10.6071/M3J67R) and δ13CH4 data (https://datadryad.org/stash/dataset/doi:10.6071/M3NX08) are available in Dryad. Source data are provided with this paper.

Field-specific reporting

Please select the one below that is the best fit for your research. If you are not sure, read the appropriate sections before making your selection.

☐ Life sciences ☐ Behavioural & social sciences ☒ Ecological, evolutionary & environmental sciences

For a reference copy of the document with all sections, see nature.com/documents/nr-reporting-summary-flat.pdf

Ecological, evolutionary & environmental sciences study design

All studies must disclose on these points even when the disclosure is negative.

Study description	Surface lake water was collected in 5 high elevation lakes. Water collected was taken back to the lab and incubated under different treatments (high-light, dark conditions and with the addition of a methanogenesis inhibitor BES) and methane concentrations from the headspace were measured over time. Additionally, we filtered water at the beginning and end of the experiment to analyze the microbial communities present or active at the time of sampling (16S rRNA, metagenomes and metatranscriptomes). For certain incubations we also measured and analyzed stable isotopic composition of methane gas collected.
Research sample	Water samples were collected in 2016-2018 in five high-elevation lakes in Yosemite National Park, and used in incubation experiments. At each lake, we sampled surface water (0.1m) to capture the surface water microbial communities. Samples were collected in Lukens (L), Lower Cathedral (LC), Upper Cathedral (UC), Lower Gaylor (LG), and Upper Gaylor (UG) Lakes. Experiments are denoted by lake abbreviation and sequential numbering for each incubation experiment (e.g. L1, L2, LC1, UC1, LG1, LG2 UG1, etc.) Lake water from high elevation lakes was chosen as a sample as the overall aim of this research project was to understand the role that surface water microbes in this ecosystem have in paradoxical methane production.
Sampling strategy	We collected water samples every 2-3 weeks over 3 sampling seasons (summer-fall of 2016, 2017 and 2019). We sampled these lakes 19 times and used water samples to run 19 incubation experiments, which exceeds the number of incubations in the majority of the published papers in this field of study (e.g., Grossart et al 2011, Tang et al 2014, Gunthel et al 2020); moreover, this allowed us to have a representation of temporal changes (seasonal and yearly) in surface methane concentrations and microbial communities in these lakes. At each field sampling time, we collected enough water (about 5 litters) to be able to collect triplicate samples at multiple timepoints from each treatment. Triplicate samples are necessary to capture any variation across replicates, and multiple timepoints are needed to capture nonlinear behavior over time. Water samples were minimally disturbed and incubations started as soon as we returned from field collection.
Data collection	Data were recorded in field and lab notebooks by Elisabet Perez Coronel while in the field sites and transfered into spreadsheets.
Timing and spatial scale	Sampling timing was dictated by field work accessibility due to weather conditions (this is because the roads are close and sites are inaccessible due to snow and ice from Nov-Jun). Samples were collected every 2-3 weeks during the summer and fall (July - November) seasons of 2016, 2017 and 2018 to have a representation of temporal changes in methane and microbial communities. Samples used in the experiment were a mix of littoral and limnetic lake water sampled at 0.1 m.
Data exclusions	No data were excluded
Reproducibility	No experiments were directly reproduced, as our aim was to apply the same experimental setup to different lakes and different sampling times (in order to capture variation and allow us to identify particular processes).
Randomization	Lake water was added into bottles that were characterized by a color tag for each treatment type; at the time of measurements, bottles were selected at random from those within the same color grouping.
Blinding	All collected samples were assigned random sample numbers, and these sample numbers were used throughout laboratory and statistical analyses until comparative analyses were necessary for each treatment group. Blinding was not possible during experiments, as all treatments needed to be kept under different conditions which were self explanatory of which treatment was which (i.e., in the dark or in a growth chamber).

Did the study involve field work? ☒ Yes ☐ No

Field work, collection and transport

Field conditions	At each location and sampling time we collected lake water for incubations and measured temperature and dissolved oxygen concentrations (using a ProODO YSI probe) and methane concentrations (samples taken on site but analyzed later in gas chromatograph). Temperature, dissolved oxygen and methane concentrations measurements were taken at the time of the collection as they were crucial to determine the baseline conditions of the system we were trying to reproduce in the incubations and to accurately calculate methane concentration on water. Additionally, we collected water samples for further analysis of nitrite, nitrate, ammonium, and phosphate. Conditions were consistently sunny during summer in the High Sierra.
Location	Samples were collected in 5 lakes in Yosemite National Park at 0.1 m depth:<br>Lukens Lakes: Elevation: 2489 m, Lat: 37.8598, Lon: 119.6160<br>Lower Cathedral Lake: Elevation: 2815 m, Lat: 37.8450, Lon: 119.4241<br>Upper Cathedral Lake: Elevation: 2905 m, Lat: 37.8395, Lon: 119.4154<br>Lower Gaylor Lake: Elevation: 3115 m, Lat: 37.9093, Lon: 119.2862

	Upper Gaylor Lake: Elevation: 3185 m, Lat: 37.9223, Lon: 119.2673
Access & import/export	Samples were collected in Yosemite National Park lakes following regulations established by research permits YOSE-2016-SCI-0118, YOSE-2017-SCI-0104 and YOSE-2018-SCI-0091
Disturbance	None

Reporting for specific materials, systems and methods

We require information from authors about some types of materials, experimental systems and methods used in many studies. Here, indicate whether each material, system or method listed is relevant to your study. If you are not sure if a list item applies to your research, read the appropriate section before selecting a response.

Materials & experimental systems

n/a	Involved in the study
☒	☐ Antibodies
☒	☐ Eukaryotic cell lines
☒	☐ Palaeontology and archaeology
☒	☐ Animals and other organisms
☒	☐ Human research participants
☒	☐ Clinical data
☒	☐ Dual use research of concern

Methods

n/a	Involved in the study
☒	☐ ChIP-seq
☒	☐ Flow cytometry
☒	☐ MRI-based neuroimaging
